# Development of a 3D Printing Liquid Crystal Display (LCD)-Assisted Micromolding Methodology for Custom Fabrication of Polymeric Microneedles Using Experimental Design

**DOI:** 10.3390/pharmaceutics17121571

**Published:** 2025-12-05

**Authors:** Lefkothea Antonara, Dimitrios M. Rekkas, Natassa Pippa, Paraskevas P. Dallas

**Affiliations:** Section of Pharmaceutical Technology, Department of Pharmacy, School of Health Sciences, National and Kapodistrian University of Athens, 15784 Athens, Greece; lefkanto@pharm.uoa.gr (L.A.); rekkas@pharm.uoa.gr (D.M.R.)

**Keywords:** liquid crystal display, polymeric microneedles, ropinirole, design of experiments, transdermal delivery, personalization

## Abstract

**Background/Objectives**: Polymeric microneedles are an innovative drug delivery form combining the benefits of both transdermal and intravenous administration. However, their practical application is limited by fabrication challenges. To address this, the study explores a novel approach for the rapid, precise, and customized production of polymeric microneedles of diverse geometries by utilizing Liquid Crystal Display (LCD) 3D printing technology, marking the first reported use of this technique for microneedle mold fabrication. **Methods**: LCD 3D printing technology was applied to prepare resin biocompatible microneedle molds. The method developed was optimized by identifying and controlling the critical process parameters (CPPs) through implementing statistical experimental design techniques within the Quality by Design regulatory framework for pharmaceutical development. The optimized molds were subsequently utilized to produce polyvinyl alcohol microneedles with customized shapes and geometries. Representative designs were then loaded with Ropinirole Hydrochloride as a model drug and evaluated in relation to their morphology, drug content, skin insertion depth, and permeability. **Results**: The application of a Central Composite Design identified layer height and exposure time as the critical process parameters affecting mold fabrication. The optimized design space enabled the selection of printing conditions that maximized dimensional accuracy. Employing these optimum LCD 3D printing parameters, microneedles of various shapes and dimensions were successfully fabricated, exhibiting highly dimensional accuracy. Additionally, tuning skin permeability was proven to be feasible by adjusting microneedle geometry. **Conclusions**: This work demonstrates the successful use of LCD 3D printing technology in producing biocompatible molds for customized microneedle fabrication, facilitating the development of transdermal delivery systems with personalized drug permeation profiles.

## 1. Introduction

Microneedles are a technological platform in micron-sized scale, ideal for the penetration of the stratum corneum. They can be used for resolving the challenges of percutaneous delivery and absorption. There is an increasing number of studies in the literature, as well as patents in the field of design and development pertaining to microneedles for drug delivery purposes. Solid, hollow, coated, hydrogel-forming, and dissolvable are the main classes of microneedles, each one exhibiting advantages and limitations in manufacturing and drug delivery [1]. Solid and coated microneedles exhibit excellent mechanical strength and very good skin-puncturing capability, while hollowed rapid- or burst-release and dissolving ones have controlled release properties. Hydrogel-forming microneedles exhibit high loading efficiency and benefit from a simple preparation process.

Polymers are the most common excipients used for the fabrication of microneedles. Biopolymers such as poly(lactic-*co*-glycolic acid), chitosan, hyaluronic acid, fish gelatin, and others are the main components of this drug delivery platform. Most of them are biocompatible and biodegradable, with easy manufacturing processes and scalable production [2]. Another important technological advantage of polymeric microneedles is the sustained or controlled release of the encapsulated active pharmaceutical ingredients (APIs), while the chemical versatility of polymers can face several limitations related to APIs in their Absorption, Distribution, Metabolism, Excretion, and Toxicology (ADME(T)) profile. The improved patient compliance due to the minimal invasiveness and pain-free procedure has led scientists to use polymeric microneedles for drug delivery purposes. Recent advances, such as using computational modeling and artificial intelligence in microneedle fabrication and development, have led to optimization of the physicochemical and mechanical properties with a high degree of accuracy and precision in microneedles [3].

Furthermore, it should be noted that challenges are still present regarding the preparation and characterization of microneedles. The stability of the API incorporated into a microneedle, as well as its physicochemical stability, is of particular importance and should be evaluated during the preformulation studies. The mechanical strength and stability of microneedles, both physicochemical and biological, also represent another technological challenge [4,5]. The scale-up, quality control during manufacturing, the batch-to-batch variability, and the regulatory gaps during the development and the preparation of the technical dossier of the final pharmaceutical product are multidisciplinary challenges [5]. Last but not least, the packaging of the microneedles and the cost are still issues hindering the rapid clinical translation of microneedle-based products [4]. The clinical adaptation and post-marketing evaluation are considered regulatory challenges addressed in the recent literature.

Several methods have been developed for the preparation of polymeric microneedles, including drawing lithography, photolithography, droplet-born air blowing, electro drawing, continuous liquid interface production, micromolding, etc. Among these, micromolding is considered the most widely used due to its time and cost efficiency, high reproducibility, scalability, and the overall simplicity of the fabrication process. The procedure generally includes five steps, beginning with the preparation of high-quality master templates [6]. In terms of simplifying the preparation process, research studies such as the one previously presented [7] introduced the concept of the direct fabrication of microneedle molds using 3D printing techniques. Based on this and in terms of enhancing the versatility of the developed method, resin printed molds were alternatively prepared from flexible materials to provide enhanced structural detail while allowing for the fabrication of microneedles of tunable geometry and dimensions. This approach complements earlier work by expanding the range of accessible mold characteristics while maintaining the advantages offered by 3D printing–assisted micromolding fabrication.

Ropinirole is a nonergoline dopamine D2 agonist with proven efficacy in the treatment of Parkinson’s disease at the early and advanced stages. Several drug delivery systems have been developed for the delivery, targeting, and controlled release of ropinirole and ropinirole hydrochloride via numerous administration routes, including the nasal, transdermal, and ocular routes [8,9,10,11]. Namely, Azeem et al. [8] developed a nanoemulsion gel for the transdermal administration of ropinirole with enhanced anti-Parkinson activity. Kale et al. [12] developed a delivery system for transdermal permeation of ropinirole hydrochloride using microneedles from a polymeric blend of polyvinyl alcohol (PVA) and polyvinyl pyrrolidone (PVP). According to their results, the combination of modulated iontophoresis and microneedles exhibited significantly higher penetration of the encapsulated API compared to modulated iontophoresis alone.

The aim of this study is to design and develop microneedles composed of polyvinyl alcohol. Design of Experiments (DoE) was used for the microneedle mold fabrication process. Several techniques were applied for the physicochemical characterization of the prepared systems. Ex vivo permeation studies showed the interrelationship between the permeation behavior of the APIs associated with the shape of the microneedles. Ropinirole HCl was used as a model API in order to evaluate the drug-loading capacity of the prepared formulations. To the best of the authors’ knowledge, this is the first time Liquid Crystal Display (LCD) three-dimensional (3D) printing methodology was implemented for the preparation of adaptable microneedle mold geometries. 

## 2. Materials and Methods

### 2.1. Materials

3Dresyn Bioflex D60 MB Monomer was obtained from 3Dresyns^®^ (Barcelona, Spain), polyvinyl alcohol Emprove^®^ PVA 40-88 from Merck (Darmstadt, Germany), and Parafilm^®^ from Sigma-Aldrich (Taufkirchen, Germany). Ropinirole hydrochloride (RopHCl) was purchased from Ind-Swift Laboratories Ltd. (Mohali, India). The polyethylene backing film (3M™ Scotchpak™ 9733) was provided by 3M (St. Paul, MN, USA), while the Primeliner^®^ 75 μm C/PET C1S liner was obtained from Loparex Paper Products Ltd. (Hammond, Germany), and Duro-Tak^®^ 87-4287 pressure sensitive adhesive from Henkel (Düsseldorf, Germany). Full-thickness porcine abdominal skin was freshly excised from animals at a local slaughterhouse. All other chemicals and solvents used in this study were of analytical grade.

### 2.2. High-Performance Liquid Chromatography (HPLC) Method for Quantitative Analysis of RopHCl

Quantification of ropinirole hydrochloride (RopHCl) samples was carried out using reversed-phase High-Pressure Liquid Chromatography (HPLC) equipped with UV detection. The procedure followed was in accordance with the USP-NF monograph for ropinirole tablets (Monograph No. M73898). Specifically, chromatographic separation was performed on an Accucore™ XL C8 column (250 mm × 4.6 mm, 4 µm particle size; Thermo Fisher Scientific, Waltham, MA, USA) under isocratic conditions at a detection wavelength of 250 nm. The mobile phase consisted of acetonitrile, methanol, and an aqueous buffer (7:3:40, *v/v/v*). The buffer was prepared by dissolving 3.85 g/L of ammonium acetate in water and adjusting the pH to 2.5 using phosphoric acid. The flow rate was set at 1.0 mL/min, with an injection volume of 50 μL, and the analyses were conducted at ambient temperature. RopHCl was eluted at approximately 6 min. Calibration curves over an appropriate concentration range were constructed using least-squares linear regression to enable accurate quantification of the samples. Chromatographic data was processed using ChromQuest^®^ Chromatography Data System, version 5.1 (Thermo Quest, Waltham, MA, USA).

### 2.3. Optimization of Microneedles’ Mold Fabrication Process via Experimental Design

Cylinder-shaped biocompatible resin molds were fabricated using a laboratory-scale Phrozen Sonic 4K LCD 3D printer (PHROZEN TECH Co., Ltd., Hsinchu, Taiwan). Three-dimensional models were designed in Autodesk Fusion© (v2602.1.25), and the printing parameters were customized using Chitubox software (v1.9.3), generating the respective .stl and .ctb files. Each mold, with an internal diameter of 37.0 mm and a wall thickness of 2.0 mm, was designed to contain four (4) microneedle arrays, allowing them to fit the centrifugation test tubes. 

**Figure 1:**
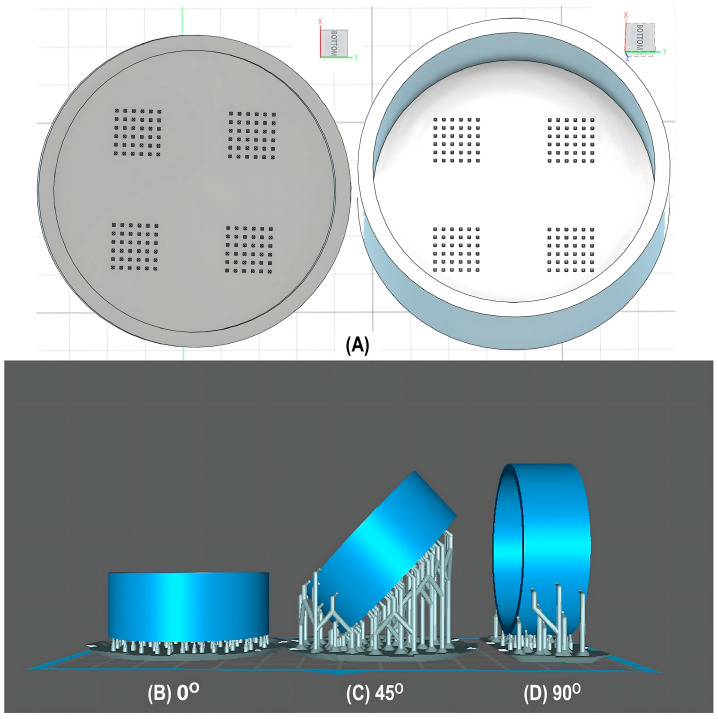
(**A**) Computer-Aided Design (CAD) of microneedle molds with Autodesk Fusion© (v2602.1.25) and graphical representation of the microneedle molds at (**B**) horizontal (0°), (**C**) inclined (45°), and (**D**) vertical (90°) orientations as generated in the Chitubox software (v1.9.3).

For the initial evaluation of the 3D printing performance, pyramid-shaped microneedles were selected as reference structures. Arrays were designed with a 6.0 mm square layout containing a number of 36 microneedles (6 × 6). The individual microneedle cavities acquired a base and height of 400 μm and 750 μm, respectively. The aforementioned dimensions were selected based on the well-established acceptable ranges reported in previous studies [13]. A graphical representation of the microneedle array geometry is demonstrated in Figure 1A.

In terms of initiating the evaluation of 3D dimensional accuracy, print quality in three different orientations was examined: horizontal (0°), inclined (45°) and vertical (90°) (Figure 1B–D). As supported by previous literature findings, printing accuracy and surface smoothness are significantly affected by printing orientation [14]. Based on the extracted results, the orientation demonstrating the best overall performance was selected for the subsequent process optimization studies.

Following this, a risk-assessment analysis was carried out to identify the potential factors affecting 3D printing accuracy. From this preliminary evaluation and after searching the literature [15,16], printing speed, layer height, and exposure time were identified as potential critical process parameters (CPPs). To optimize these variables, a Face-Centered Central Composite Design (FCCD) with *α* = 1 was carried out. The factor levels were defined, taking into account the working range of the 3D printer, while focusing on achieving fine detail parts, due to the small size of the microneedles. Additionally, a design constraint was introduced between the factors (B) and (C), given that thicker layers require correspondingly longer exposure times. The constraint equation was extracted based on the worst-case scenario under which the maximum layer height 50 μm required a minimum of 7.5 s exposure to light to allow for curing. This approach ensured that, under all experimental combinations, polymerization of the resin layers takes place. The parameters of the optimization design are summarized in Table 1.

Overall, seventeen (17) experimental runs with three (3) replicates at the center point were carried out. The matrix of the experimental design for the optimization of the 3D printing process factors is presented in Table 2. To determine the responses, the parameters examined were the surface area of mold cavities (mm^2^) along with microneedle height (mm). The latter were measured utilizing a Kern OZL 466 Trinocular (KERN & SOHN GmbH, Balingen, Germany) stereomicroscope equipped with a C-mount camera USB 2.0 ODC 825 (*n* = 10). Photos were captured with the software ToupView (version 4.11.19728.20211022; ToupTek, China) and analyzed with ImageJ 1.54p (NIH, Bethesda, MD, USA).

### 2.4. Microneedle Mold Preparation Process

Biocompatible molds for the micromolding of polymeric microneedles were prepared utilizing a laboratory-scale Phrozen Sonic 4K LCD 3D printer, with the optimum set of parameters identified from the experimental design. The rest of the settings were kept constant across all experimental runs and are presented in Table 3.

Upon completion of the printing process, the molds were left on the build platform for 30 min to allow excess uncured resin to drain. Afterwards, the molds were carefully removed, immersed in an isopropyl alcohol bath, and sonicated for 15 min. During cleaning via sonication, the interior cavity of the molds was positioned facing the base of the sonicator [17] to ensure resin removal. After cleaning, pressurized air was applied to the same part to remove the diluent and promote drying. To post-cure the 3D printed biocompatible molds and polymerize any uncured resin residues, the Anycubic Wash & Cure 2.0 system (Anycubic, Shenzhen, China) was utilized, exposing the parts to 405 nm for 20 min. After that, the molds were ready to be used for the microneedle fabrication process.

### 2.5. Microneedle Preparation Process

RopHCl-loaded polymeric microneedles were fabricated using a micromolding technique with polyvinyl alcohol (PVA) and polysorbate-80 (Tween-80) as the component materials. The formulation of the solution consisted of 10% (*w/w*) PVA 40-88, 1.5% (*w/w*) Tween-80, and 1% (*w/w*) active ingredient (API). The preparation process was carried out in accordance with the following procedure. In a glass container of appropriate volume, 70% of the total required deionized water was added and heated to 90 °C. PVA was then gradually added under continuous stirring with an overhead stirrer until fully dissolved. The solution was then removed from the heat and left to cool down to room temperature (Phase A). In a separate glass container, 20% of the total water amount and the plasticizer polysorbate-80 were added and mixed with a magnetic stirrer, until a homogeneous solution was obtained. RopHCl was then incorporated into the plasticizer solution and stirred until complete dissolution (Phase B). Phase B was subsequently added to Phase A, and the combined solution was mixed thoroughly to ensure homogeneity. The microneedle films were then prepared using the 3D printed molds. In more detail, 4.5 g of the solution was dispensed into the 3D printed molds and subjected to centrifugation at 4000 rpm for 10 min to ensure complete filling of the cavities. Subsequently, they were left to dry at 40 °C overnight and then were manually demolded by hand. Finally, the arrays were die-cut into circular films with an area of 1.27 cm^2^, each containing approximately 3 mg of RopHCl per cm^2^.

### 2.6. Performance Evaluation of Printing Process Across Multiple Shapes and Dimensions

Enhancement of skin permeation through the application of microneedle arrays can be effectively tuned by adjusting microneedle geometry and dimensions. In particular, microneedles with greater volume are expected to form higher-capacity microchannels, thereby facilitating the permeation of active ingredients through the skin. Based on the above, the ability of the previously described optimized technology to accurately fabricate these microstructures had to be further investigated. For this purpose, a literature search was carried out to identify the most frequently fabricated microneedle shapes (Table 4). 

Afterwards, the performance of the 3D printed-assisted micromolding technique was evaluated in relation to the reproducibility of these shapes and modified designs, across the minimum and maximum dimensional acceptable range reported. Specifically, microneedle height typically ranges from 100 to 1000 μm [13], and width from 150 to 500 μm, while the diameter of the microneedle tip may vary from 1 to 50 μm. Initial screening trials, using different shapes, indicated no dimensional limitations in the upper set level. However, at the lower limits, i.e., 100 μm and 150 μm, cavities were inadequately formed. As a result, the lower limit of 300 μm was set for both microneedle height and width. All microneedles were designed as sharp-tipped using the 3D computer-aided software.

With regard to the microneedle array density, a constant interspacing distance of 300μm was set for all shape trials carried out. However, to further evaluate accuracy at minimal interspacing spaces, the (a)-aspect ratio criterion introduced by several publications [18,19] was considered in square pyramid microneedles at the lowest dimensional end. Parameter (a) is calculated through the following equation (Equation (1)) and ranges from 2.7 to 12, with an optimum value of 4. At values below 2.7, the microneedle array packing is considered too dense, affecting skin puncture due to the ‘’bed of nails’’ effect.

(a) = (Interspacing Distance + Base Width)/(1⁄2 Base Width)(1)

A summarized representation of the trials carried out across different geometries and dimensions is demonstrated in Table 4.

**Table 4:** Summary of microneedle geometries and dimensions investigated during printing process performance.

Geometry	Dimensions	3D Design	Reference(s)
Cone	**Base Diameter**: 300 μm (lower limit) **Height**: 300 μm (lower limit) **Interspacing Distance**: 300 μm	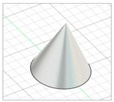	[20,21,22]
	**Base Diameter:** 500 μm (upper limit) **Height:** 1000 μm (upper limit) **Interspacing Distance:** 300 μm	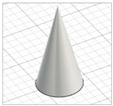	
Inclined cone (tip at the edge)	**Base Diameter:** 300 μm (lower limit) **Height:** 650 μm (medium level) **Interspacing Distance:** 300 μm	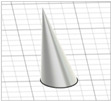	Modification of conical ones—no references found
Triangular pyramid	**Base Height:** 300 μm (lower limit) **Height:** 300 μm (lower limit) **Interspacing Distance:** 300 μm	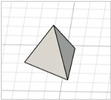	[22,23]
	**Base Height:** 500 μm (upper limit) **Height:** 1000 μm (upper limit) **Interspacing Distance:** 300 μm	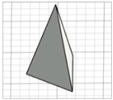 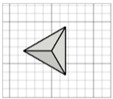	
Inclined triangular pyramid (tip at the edge)	**Base Height:** 300 μm (lower limit) **Height:** 650 μm (medium level) **Interspacing Distance:** 300 μm	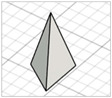 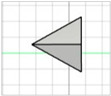	Modification of triangular pyramid ones—no references found
Square pyramid	**Base Width:** 300 μm (lower limit) **Height:** 300 μm (lower limit) **Interspacing Distance:** 105 μm (the minimum value of the aspect ratio must be 2.7)	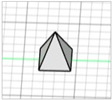	[20,24,25,26]
	**Base Width:** 300 μm (lower limit) **Height:** 300 μm (lower limit) **Interspacing Distance:** 300 μm		
	**Base Width:** 500 μm (upper limit) **Height:** 1000 μm (upper limit) **Interspacing Distance:** 300 μm	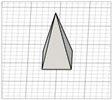	
Square pyramid with angled edges	**Base Width:** 300 μm (lower limit) **Height:** 300 μm (lower limit) **Interspacing Distance:** 300 μm	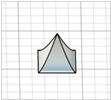	Modification of square pyramid ones—no references found
	**Base Width:** 500 μm (upper limit) **Height:** 1000 μm (upper limit) **Interspacing Distance:** 300 μm	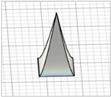	
Hexagonal-based pyramid	**Base Width:** 300 μm (lower limit) **Height:** 300 μm (lower limit) **Interspacing Distance:** 300 μm	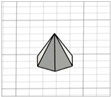	[22]
	**Base Width:** 500 μm (upper limit) **Height:** 1000 μm (upper limit) **Interspacing Distance:** 300 μm	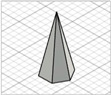	
Beveled-tip	**Base Diameter:** 300 μm (lower limit) **Height:** 300 μm (lower limit) **Bevel angle:** 60° **Interspacing Distance:** 300 μm	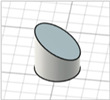 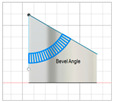	[20,22,27]
	**Base Diameter:** 500 μm (upper limit) **Height:** 1000 μm (upper limit) **Bevel angle:** 45° **Interspacing Distance:** 300 μm	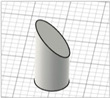	
Tapered-cone	**Base Diameter:** 300 μm (lower limit) **Height:** 300 μm (lower limit); the individual height of the cone and cylinder equals 150μm **Interspacing Distance:** 300 μm	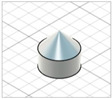	[20]
	**Base Diameter:** 500 μm (upper limit) **Height:** 1000 μm (upper limit); the individual height of the cone and cylinder equals 500μm **Interspacing Distance:** 300 μm	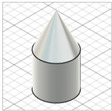	
Obelisk	**Base Width:** 300 μm (lower limit) **Height:** 300 μm (lower limit); the individual height of the square pyramid and base equals 150μm **Interspacing Distance:** 300 μm	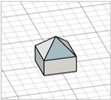	[23,28]
	**Base Width:** 500 μm (upper limit) **Height:** 1000 μm (upper limit); the individual height of the square pyramid and base equals 500μm **Interspacing Distance:** 300 μm	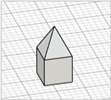	
Modified pyramid	**Base Width:** 300 μm (lower limit) **Height:** 300 μm (lower limit) **Interspacing Distance:** 300 μm	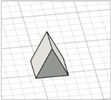	[29]
	**Base Width:** 500 μm (upper limit) **Height:** 1000 μm (upper limit) **Interspacing Distance:** 300 μm	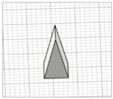	
Arrow-like	**Base Width:** 300 μm (lower limit) **Height:** 300 μm (lower limit); the individual height of the modified pyramid and base equals 150 μm **Interspacing Distance:** 300 μm	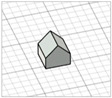	[30]
	**Base Width:** 500 μm (upper limit) **Height:** 1000 μm (upper limit); the individual height of the modified pyramid and base equals 500μm **Interspacing Distance:** 300 μm	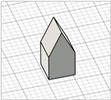	

Afterwards, the fabricated microneedle arrays of various geometries were characterized with respect to their dimensional accuracy using a Kern OZL 466 Trinocular (KERN & SOHN GmbH, Balingen, Germany) stereomicroscope equipped with a C-mount camera USB 2.0 ODC 825 and a Hitachi SU3800 Scanning Electron Microscope at various magnifications at 15 kV. (Hitachi, High Tech Corporation, Tokyo, Japan).

### 2.7. Performance Study of Microneedle Designs for Adjusting Transdermal Permeation

Microneedles represent an emerging platform for the enhancement of skin permeation, developed to mechanically surpass the stratum corneum barrier, which is the primary obstacle to transdermal drug delivery. The degree of drug permeation facilitated by microneedles is strongly influenced by microchannel formation in the skin, with the characteristics of the latter determined by the microneedle geometry and dimensions [31,32,33]. The ability of the 3D printing process to accurately reproduce the most commonly used microneedles was evaluated as described in the previous section.

Based on this, the impact of microneedle geometry on the permeation of the active pharmaceutical ingredient was evaluated through the study of three representative microneedle designs—square pyramid, beveled-tip and obelisk. Each design was fabricated with dimensions set at the intermediate level of the studied dimensional range (Table 5). The microneedles obtained were then characterized in relation to their drug content, insertion depth, and permeation behavior.

### 2.8. Preparation of the Support Patch for the Diffusion Studies

In terms of stabilizing the polymeric microneedle arrays on the skin surface, a non-loaded (placebo) transdermal patch was applied as supportive overlay. The preparation process began by placing a release liner on a clean, flat surface, with the siliconized (non-stick) side facing upward. The pressure-sensitive adhesive Duro-Tak^®^ 87-4287 was then applied at the one edge of the release liner and drawn uniformly using a 10-mil casting knife under a single, continuous motion. The adhesive layer was then dried in an oven at 70 °C for 30 min to allow for solvent evaporation and left to cool down to reach room temperature. Following this, the backing film was applied with a roller to form a laminate structure without wrinkles and air bubbles. Circular disks of 2 cm^2^ surface area were die-cut from the laminate and implemented as support patches for the microneedle application.

### 2.9. Microneedle Array Characterization

Microneedles have a long-standing presence in the pharmaceutical field as medical devices; however, there have been no universally established regulatory standards to verify microneedle quality. To address this gap, several methodologies have been proposed in the literature, including dimensional analysis through visual inspection, evaluation of insertion depth using in vitro and/or in vivo models, and quantification of the active pharmaceutical ingredient, as well as in vitro release and permeation studies [34]. In this study, some of these techniques were implemented to characterize the performance and quality of the extracted arrays.

#### 2.9.1. Microscopic Inspection of Microneedle Arrays Through Stereomicroscope 

A Kern OZL 466 Trinocular (KERN & SOHN GmbH, Balingen, Germany) stereomicroscope equipped with a C-mount camera USB 2.0 ODC 825 was utilized for the morphology observation of the microneedle arrays at appropriate angle and magnification. The photos captured with software ToupView (version 4.11.19728.20211022; ToupTek, China) were analyzed with ImageJ 1.54p (NIH, Bethesda, MD, USA) to determine dimensional accuracy for each of the studied geometries prepared (*n* = 10).

#### 2.9.2. Insertion Depth Determination Studies

One important aspect in evaluating the performance of microneedles is the determination of the microneedle insertion depth. In general, human or porcine skin samples are considered the most suitable choice; however, they present several drawbacks including heterogeneity, limited stability and challenges in sourcing. On the contrary, artificial membranes can be widely used in quality control (QC) routine analysis or for screening purposes during the early development stages [35]. One such case is Parafilm^®^ M, a commercially available polymeric film, consisting of a hydrocarbon wax and polyolefin blend. This material can simulate the mechanical and elastic properties of skin, enabling estimation of MN insertion depth. The study was carried out by manually applying the microneedle arrays for 30 s on the surface of a Parafilm^®^ sheet folded to form an eight-layer film. Visual inspection of each layer was then performed using a Kern OZL 466 Trinocular (KERN & SOHN GmbH, Balingen, Germany) stereomicroscope equipped with a C-mount camera USB 2.0 ODC 825. The final insertion depth was determined by multiplying the thickness of each layer (approximately 127 μm as per the supplier’s specifications) by the number of pierced layers.

#### 2.9.3. Quantitative Determination of Ropinirole Hydrochloride in Microneedle Arrays

Microneedle circular films of 1.27 cm^2^ in surface area were die-cut and transferred to 100 mL volumetric flasks in triplicate. The films were then dissolved with the addition of water and agitated until complete dissolution. Sonication was performed when necessary to facilitate the process. For sample analysis, the previously described HPLC method was implemented, while all experiments were performed in duplicates (*n* = 2).

#### 2.9.4. Ex Vivo Permeation Studies

The ex vivo permeation studies were carried out using vertical modified amber glass Franz diffusion cells with a receptor volume of 6.275 mL and a diffusion surface of 0.636 cm^2^. The skin tissue used was porcine non-scolded abdomen full-thickness skin obtained from a local slaughterhouse [36]. The latter was isolated by removing the subcutaneous fat tissue with a scalpel.

**Figure 2:**
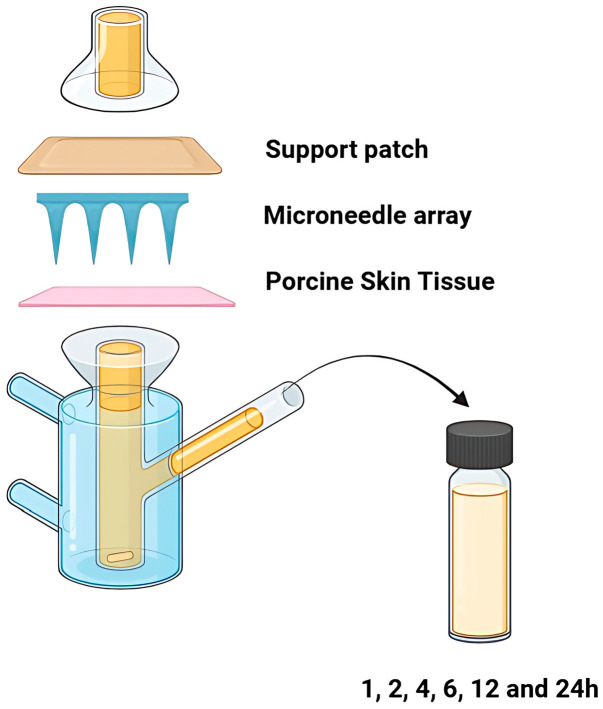
The graphical representation of the experimental set-up of the ex vivo permeation studies.

The tissue was cut and positioned over the diffusion surface area, with the stratum corneum facing the donor compartment. To enhance adhesion and simulate physiological transepidermal water loss (TEWL), the membrane was pre-wetted with 20 μL of distilled water. Microneedle arrays were then manually applied to the center through thumb force for 30 s, as described in the literature [37]. Stabilization of the arrays on the skin surface was guaranteed by the application of the support patch as an overlay, and then the whole assembly was secured between the two compartments using metallic clamps (Figure 2).

The receptor chamber was filled with phosphate buffer pH 7.4, thermostated at 32 ± 0.5 °C, using a heated water bath and stirred continuously at 600 rpm with a magnetic stirrer to ensure sink conditions. During the experiment, five replicates were performed per formulation (*n* = 5). At predetermined time intervals (1, 2, 4, 6, 12, and 24 h), the entire amount of the receptor medium was withdrawn and replaced with an equal volume of fresh medium [7]. The samples collected were analyzed via the previously described HPLC methodology.

## 3. Results

### 3.1. Liquid Crystal Display (LCD) 3D Process Design

During the initial 3D process development phase, three different orientations were evaluated—0°, 45°, and 90°—as detailed in Section 2.3. Among these, the horizontal orientation (0°) yielded the best performance, combining the shortest printing time, high dimensional accuracy, and smoothest surface finish. In contrast, in the vertical one (90°), a collapse of the interior design geometry of the cavities was observed, probably due to a lack of supports, with high observed dimensional deviations. While the addition of support structures could minimize this, their addition was not feasible due to their small size, making them difficult to post-process and remove. Regarding the inclined position (45°), the printed parts showed satisfactory dimensional accuracy results; however, they exhibited an uneven surface, which could probably be attributed to the stair-stepping effect. Based on these findings, the horizontal orientation was selected as the optimal orientation for fabricating mold structures.

The FCCD was performed to characterize the design space and identify the optimum 3D printing working parameters. Overall, seventeen (17) experiments were performed. The results of the experimental design for the two responses studied, i.e., the surface area of the mold cavities and the microneedle height, are presented in Table 6.

Subsequently, analysis of variance (ANOVA) was performed to analyze the extracted data and identify the relationships between the input parameters and the responses measured.

#### 3.1.1. Analysis of Response 1: Surface Area of Mold Cavities (mm^2^)

Based on the results of Table 4, the values of Response 1 ranged from 0.094 to 0.167 mm^2^, with the relative standard deviation (%RSD) exhibiting values from 1.6% to 9.1%, demonstrating good reproducibility. The ANOVA indicated that the Modified Quadratic Model was statistically significant (*p* < 0.05), suggesting that the input factors have a significant impact on the response evaluated (Table 7).

Among the factors, exposure time (C), printing speed (A) and the squared term of layer height (B^2^) were highlighted as the significant process parameters affecting the response. Exposure time was revealed as the factor with the highest contribution to the response (highest *F*-value), while printing speed marginally influenced the outcome, with a *p*-value close to 0.05 threshold. The lack-of-fit *p*-value lay well above 0.05, indicating a good fit of the model to the experimental data, while adjusted *R*-squared and predicted R-squared demonstrated the predictive capability of the extracted model. 

**Figure 3:**
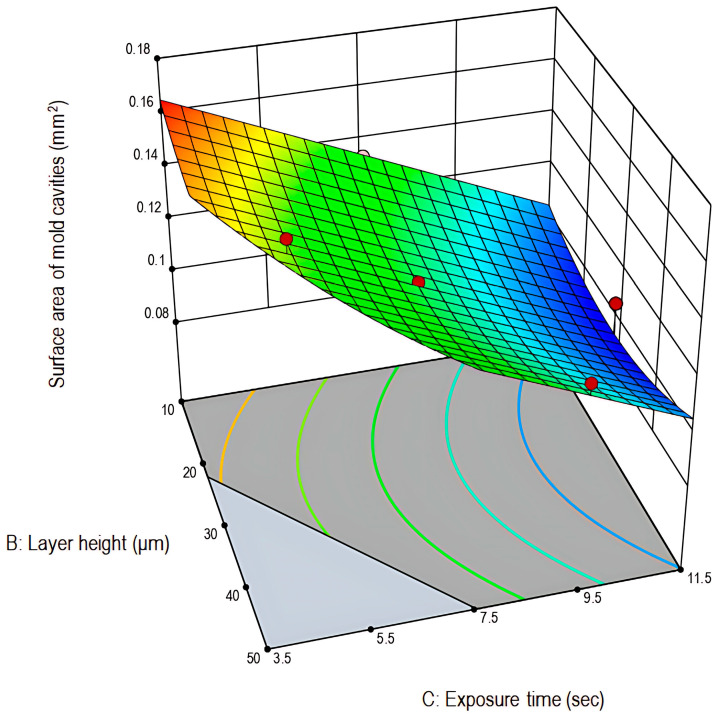
Three-dimensional graph plot for the surface area of the mold cavities (mm^2^) in relation to layer height (μm) and exposure time (s) (Stat-Ease 360^®^).

As illustrated in the 3D-graph plot (Figure 3) and in consistency with the preliminary trials and literature findings, prolonged exposure times result in light leakage due to light scattering, thus causing dimensional expansion and cavities shrinkage [38]. This over-curing phenomenon can be attributed to polymerization outside of the desired exposure field. Additionally, lower layer-height prints were characterized by their smoother surface and improved print quality. Finally, the theoretically set dimensions (0.160 mm^2^) were satisfactorily reproduced at the experimental space where low exposure time and low layer-height values were combined.

#### 3.1.2. Analysis of Response 2: Height of Microneedles (mm)

Similarly to the analysis of the Response 1: Surface area of microneedles cavities, the Response 2 values ranged from 0.102 to 0.731, with good reproducibility as indicated by %RSD values between 1.4% to 9.4%. The ANOVA demonstrated that the quadratic model was statistically significant (*p* < 0.05), suggesting that the input factors have a significant impact to the response evaluated (Table 8).

From the factors investigated, layer height (B) and exposure time (C) along with their squared terms (B^2^ and C^2^) were indicated as the critical process parameters (CPPs) influencing the height of microneedles. Again, exposure time demonstrated the highest contribution to the response (highest *F*-value), followed by the terms correlated to layer height. The lack-of-fit *p*-value was above the 0.05 threshold of 0.05, revealing the good fit of the model to the experimental data. Adjusted *R*-squared and predicted *R*-squared proximity to 1.0000 demonstrated the strong predictive capability of the selected model.

**Figure 4:**
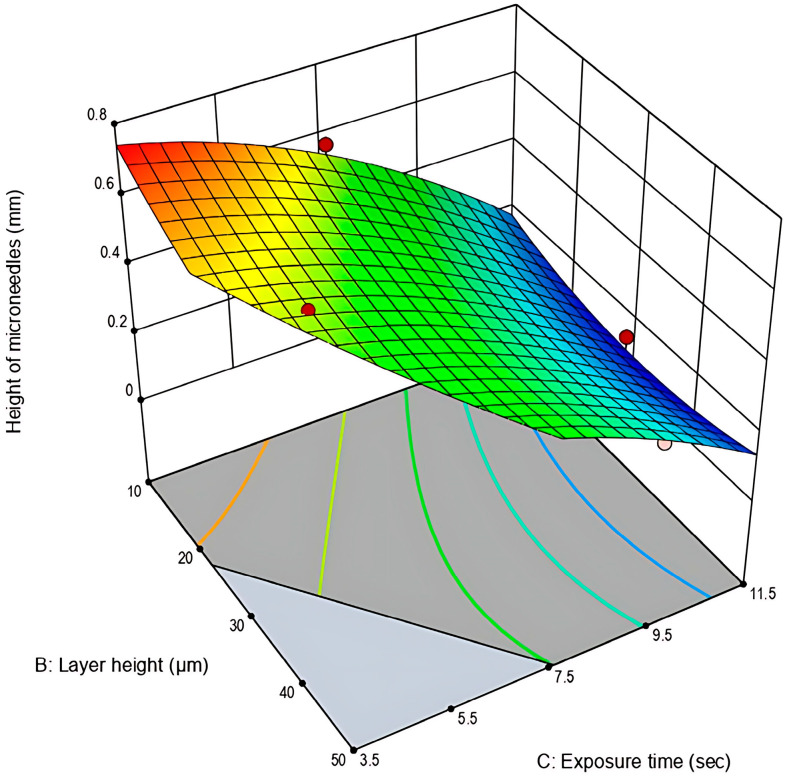
Three-dimensional graph plot for the height of microneedles (mm) in relation to layer height (μm) and exposure time (s) (Stat-Ease 360^®^).

As presented in the 3D-graph plot (Figure 4) and similarly to the results obtained for Response 1, extended exposure times result in higher print dimensions and the shrinkage of the microneedle cavities. Also, it can be observed that the target value of 0.750 mm was not reached for any of the experimental runs. This outcome was expected, as polymeric microneedles undergo shrinkage due to water evaporation up to as much as 5.0–6.0% of their original dimensions [25]. Overall, although the theoretically set height (0.750 mm) was not reached, the closest approximation occurred in the experimental space where low exposure time and layer-height values were combined.

#### 3.1.3. Optimization of the 3D Printing Parameters

The optimum combination of 3D printing process parameters was selected using the desirability function approach. Specifically, the primary objective of the current study was to maximize the height of the microneedles while targeting a surface area of 0.160 mm^2^ for the microneedle cavities. Maximizing the microneedles’ height is critical, as it is directly linked to penetration depth, while achieving the target surface of cavities is a case of process reproducibility and accuracy. 

Among the number of the proposed solutions generated by the analysis of the models, the one with 100 mm/min printing speed, 10 μm layer height, and 4.0 s exposure time was the optimal solution with a desirability function of 0.990. This high desirability value suggests that the recommended set of process parameters strongly fulfills the accuracy requirements, with minor deviations.

Subsequently, within the scope of verifying the optimized settings, microneedle molds were fabricated using these process parameters, and evaluated in relation to their dimensional accuracy. The measured microneedle height was equal to 0.708 ± 9.0 μm (94.4%), while the surface area of the cavities was 0.158 ± 9.8 mm^2^ (98.8%). Finally, both measurements fell within the 95% confidence intervals predicted by the model, confirming the accuracy and reliability of the optimization approach.

### 3.2. Performance Analysis of the 3D Printing Process Across Multiple Geometries

**Figure 5:**
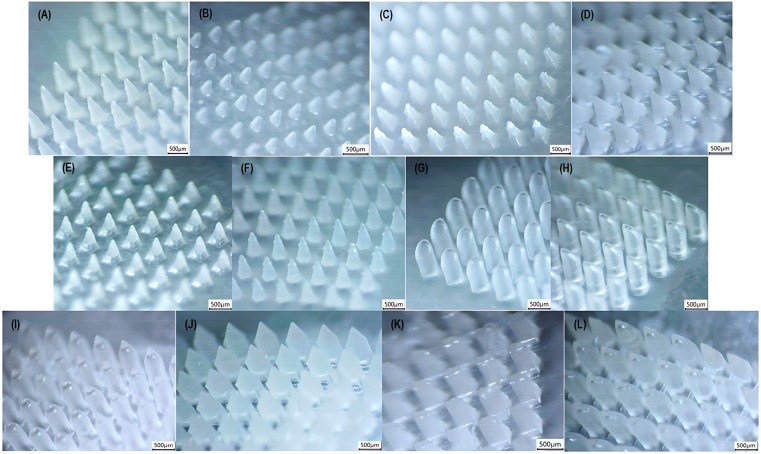
Stereomicroscopic images of microneedle shapes: (**A**) cone (upper limit); (**B**) inclined cone (tip at the edge); (**C**) triangular pyramid (upper limit); (**D**) square pyramid (upper limit); (**E**) square pyramid with angled edges (upper limit); (**F**) hexagonal-based pyramid (upper limit); (**G**) & (**H**) beveled-tip (upper limit); (**I**) tapered-cone (upper limit); (**J**) obelisk (upper limit); (**K**) modified pyramid (upper limit); and (**L**) arrow-like (upper limit); captured using a Kern OZL 466 Trinocular (KERN & SOHN GmbH, Germany) stereomicroscope.

As described previously (Section 2.6), various microneedle geometries were prepared and evaluated with regard to their dimensional accuracy to determine the versatility of the developed process across various shapes. For this purpose, biocompatible molds representing twelve (12) distinct designs were 3D printed and used for the formation of the polymeric microneedles. The extracted microstructures were visually observed and characterized (Figure 5), with the results summarized in Table 9.

The %RSD values of all measurements ranged from 0.2% to 5.6%, indicating the reproducibility of the fabrication method. Moreover, the tip diameters remained below the 50 μm threshold, ensuring skin punctuation.

According to Table 9, all examined geometries demonstrated slightly reduced dimensional trueness at the lower limit, which is likely attributed to polymer shrinkage during drying and the evaluation of smaller sizes. In contrast, differences from the theoretical values at the upper limit were considered as negligible, indicating higher dimensional accuracy at larger scale. 

Regarding shape performance, two of the evaluated shapes exhibited significantly lower dimensional values. Specifically, both the triangular and the inclined triangular pyramid were characterized by decreased dimensional accuracy at low and intermediate range. However, no issues were highlighted for this geometry at the upper limit. Similarly, the modified pyramid showed a drop in the base width feature at the lower limit, while maintaining acceptable values at the upper limit. Consequently, the developed method is considered sufficiently accurate for the fabrication of the majority of the commonly reported geometries throughout the literature.

### 3.3. Performance Study of Microneedle Designs for Adjusting Transdermal Permeation

#### 3.3.1. Microscopic Inspection of Microneedle Arrays

Polymeric microneedle arrays prepared for skin permeation studies were visually characterized in relation to their dimensional accuracy. All microneedles were removed intact from the 3D printed biocompatible molds, with acceptable deviations from the theoretical set values as demonstrated in Table 10.

#### 3.3.2. Quantitative Determination of Ropinirole Hydrochloride in Microneedle Arrays

Across all formulations evaluated for their ex vivo permeation behavior, the average RopHCl content remained within the acceptable 90.0–110.0% range. 

#### 3.3.3. Insertion Depth Determination Studies

**Figure 6:**
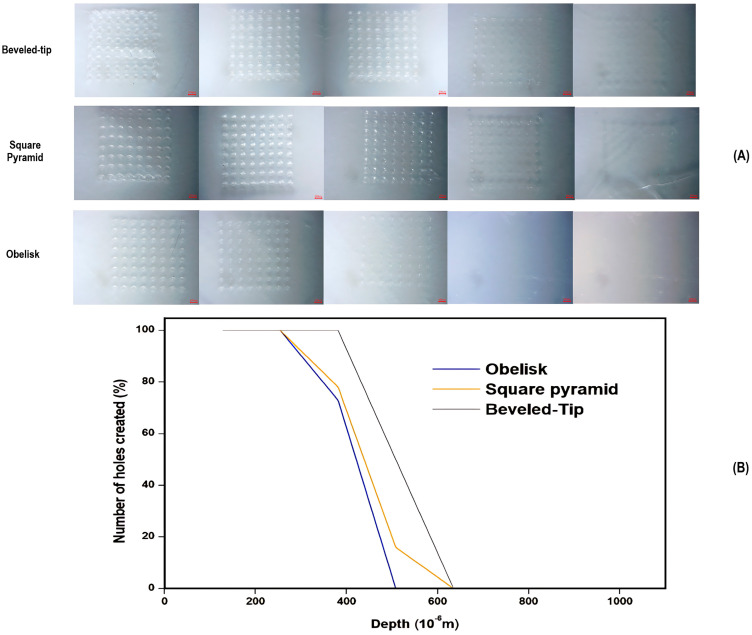
(**A**) Microscopic images of individual Parafilm^®^M layers for each of the three designs and (**B**) a graphical representation of the percentage of number of holes created in relation to the penetration depth (μm) and the number of Parafilm^®^M layers.

Microneedle skin insertion depth is one of the limiting factors in the permeation of the active substance, affected by the viscoelastic properties of the skin and the packing of the microneedle array. A dense microneedle organization could potentially lead to the “bed-of-nails” effect, thereby reducing insertion depth and consequently limiting permeation [35]. For this purpose, several models and skin simulants have been utilized to determine skin permeation at early development stages. One of these proposed model membranes is the polyolefin film of Parafilm^®^M. In this study, insertion depth was assessed according to the procedure described in the previous section. Each of the Parafilm^®^M sheets was examined microscopically to establish the percentage of holes created per layer for each of the three studied designs (Figure 6).

As per the results of Figure 6, the penetration depths of the various microneedle geometries were ranked as follows: beveled-tip > square pyramid > obelisk. For the beveled-tip and square pyramid, the maximum penetration depth was measured at approximately 508 μm, while the obelisk-shaped microneedles reached an upper limit of 381μm. These differences could be attributed to variations in microneedle volume, tip angle, and cross-sectional areas [39].

The obelisk design, although exhibiting the lowest penetration depth, was characterized by the largest volume and cross-sectional area in comparison to the rest of the geometries. On the opposite side, the beveled-tip and square pyramid shapes, with their smaller volume and cross-sectional surfaces, contribute to the deeper penetration under the application of similar forces. In general, although larger cross-sectional areas enhance mechanical endurance, they can also lead to increased tissue resistance during application. This phenomenon, called the ‘’bed-of-nails’’ effect, states that greater force distribution on larger surfaces reduces skin penetration depth values and thus skin permeation. Similarly, increased microneedle volumes elevate the resistance from the adjacent skin tissues, causing a decrease in penetration.

#### 3.3.4. Ex Vivo Permeation Studies

**Figure 7:**
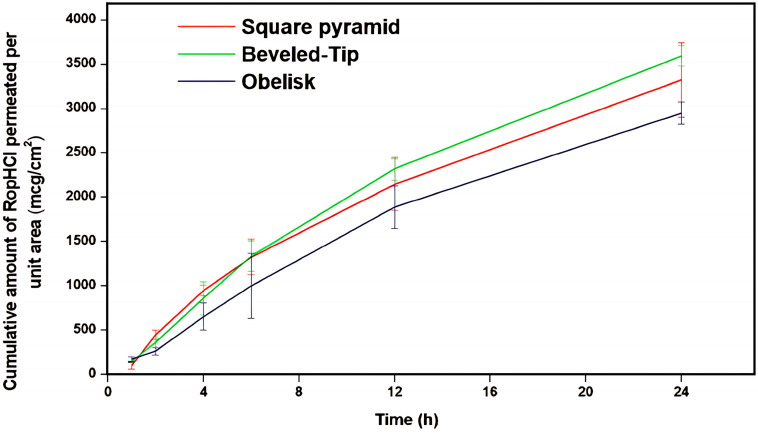
Cumulative amount of RopHCl permeated per unit area (μg/cm^2^) versus time (h) through porcine skin (*n* = 5, bars represent standard deviation).

Ex vivo skin permeation studies, assessing the three microneedle designs with porcine non-scolded abdomen full-thickness skin, were performed according to the procedure described in the previous section. The skin permeation results are summarized in Figure 7.

According to the results of Figure 7, the cumulative amount of RopHCl permeated per skin unit surface area, for the various microneedle geometries, was ranked as follows: beveled-tip > square pyramid > obelisk. Specifically, beveled-tip microneedles facilitated drug permeation followed by square pyramid and obelisk ones; with the latter demonstrating the lowest observed values. Statistical comparisons performed using a Student’s *t*-test at a 95% confidence level confirmed the significantly lower permeability observed with the obelisk geometry. Taking into account the data extracted from the insertion depth studies, it can also be concluded that the elevated performance of the beveled-tip microneedles is attributed to their superior skin punctuation, thus leading to more efficient skin permeation.

These findings are in agreement with previous studies in the literature [40,41] which similarly demonstrated the effect of microneedle design on skin permeation. Therefore, the geometry of microneedles, apart from influencing mechanical properties, has a significant impact on the formation of the skin diffusion channels, thereby influencing the overall skin flux. This geometry-dependent behavior underlines the ability to optimize the developed transdermal systems to obtain the desired bioavailability of the active substance. By altering microneedle design and insertion depth at a constant surface area, it becomes possible to tune drug-release kinetics and therapeutic profiles, while maintaining a dosage form of stable composition.

Furthermore, the trends observed in the permeation study were consistent with the results of the insertion depth study earlier presented. In more detail, microneedles achieving higher insertion depths correspond to elevated permeation rates, indicating the critical role of evaluating skin penetration depth during the early development stages.

## 4. Conclusions

An LCD 3D printing assisted method for the fabrication of biocompatible microneedle molds was successfully developed and optimized by applying the DoE principles. This technology has proven to be suitable for fabricating tunable microneedle arrays of varying shapes and dimensions. Only two out of the twelve (12) examined shapes (triangular and modified pyramid) exhibited large deviations from the theoretical set values at the lower to intermediate dimensional limit. At the upper limit, all geometries were accurately reproduced reflecting the suitability of the method to prepare shapes at larger scales. Furthermore, skin permeation studies conducted using selected microneedle shapes revealed a strong relationship between needle geometry and skin permeability. These findings reveal the ability to adjust skin permeability through geometry design, offering a promising tool for optimizing drug bioavailability in transdermal drug delivery systems, according to therapeutic needs. This can be extensively utilized in personalization cases and dose adjustment applications.

Our study is in line with the findings from the recently published data, where biocompatible polymers are used for the design and development of microneedles for the loading and modified release of active pharmaceutical ingredients. The assessment of the geometrical dimension of microneedles is of paramount importance for the effectiveness of the delivery platform [42,43,44]. The process parameters seem to play a key role in the fabrication of customized microneedle arrays. Namely, Tamez-Tamez et al. [43] found that the increase in truncation resulted in a reduction in puncturing the skin and skin permeation, respectively. The presence of multiple punctures from the microneedles increases the effective permeation area and reduces the effective diffusion path length.

In conclusion, our research follows the recent trends in the design and development of microneedles, adding the QbD approach to improve the effectiveness and contribute added value in drug delivery and targeting.

## Figures and Tables

**Table 1:** A tabular representation of the 3D printing process factors along with their levels, the responses evaluated, and the design constraint set.

Factors	Levels			Responses
	Low	Medium	High	
Printing speed (mm/min) (A)	50	100	150	Surface area of mold cavities (mm^2^) Height of microneedles (mm)
Layer height (μm) (B)	10	30	50	
Exposure time (s) (C)	3.5	7.5	11.5	
Design Constraint	Layer height ≤ 6.8 × Exposure time			

**Table 2:** Design matrix for the FCCD for the 3D printing process optimization.

Experimental Run (ER)	Factor A: Printing Speed (mm/min)	Factor B: Layer Height (μm)	Factor C: Exposure Time (s)
ER1	100	30	7.5
ER2	50	10	11.5
ER3	150	50	8.0
ER4	150	30	7.5
ER5	50	30	7.5
ER6	150	10	3.5
ER7	100	30	7.5
ER8	100	30	11.5
ER9	100	10	7.5
ER10	100	30	7.5
ER11	100	30	5.0
ER12	50	10	3.5
ER13	150	10	11.5
ER14	150	50	11.5
ER15	50	50	8.0
ER16	100	50	9.5
ER17	50	50	11.5

**Table 3:** LCD 3D printing process parameters for microneedle mold preparation.

**Printing Parameters**	**Set Value**
Bottom Layer Count	8
Bottom Exposure time (s)	120
Rest time after retract (s)	5.0
Lifting Distance (mm)	5.0
**Supports**	
Support setting	Medium
Density (%)	55.0
Angle (°)	70.0

**Table 5:** Microneedle designs and dimensions utilized for skin permeation experiments.

Microneedle Designs for Permeation Study	Dimensions
Square pyramid	**Base Width**: 400 μm **Height**: 650 μm
Beveled-tip	**Base Diameter:** 400 μm **Height:** 650 μm **Bevel angle:** 51°
Obelisk	**Base Width:** 400 μm **Height:** 650 µm the individual height of the square pyramid and base equals 325 μm

**Table 6:** Design matrix for the FCCD of the 3D printing process along with the studied responses.

Experimental Run	Factor A: Printing Speed (mm/min)	Factor B: Layer Height (μm)	Factor C: Exposure Time (s)	Response 1: Surface Area of Mold Cavities (mm^2^)	Response 2: Height of Microneedles (mm)
ER1	100	30	7.5	0.122	0.385
ER2	50	10	11.5	0.098	0.132
ER3	150	50	8.0	0.141	0.392
ER4	150	30	7.5	0.127	0.437
ER5	50	30	7.5	0.107	0.358
ER6	150	10	3.5	0.163	0.731
ER7	100	30	7.5	0.120	0.375
ER8	100	30	11.5	0.105	0.131
ER9	100	10	7.5	0.134	0.570
ER10	100	30	7.5	0.125	0.396
ER11	100	30	5.0	0.148	0.540
ER12	50	10	3.5	0.167	0.701
ER13	150	10	11.5	0.111	0.163
ER14	150	50	11.5	0.104	0.120
ER15	50	50	8.0	0.132	0.362
ER16	100	50	9.5	0.125	0.288
ER17	50	50	11.5	0.094	0.102

**Table 7:** ANOVA values for the surface area of the mold cavities (mm^2^) (Stat-Ease 360^®^).

Source	Sum of Squares	d*f*	Mean Square	*F*-Value	*p*-Value	Conclusion
Modified Quadratic Model	0.0066	4	0.0017	35.11	<0.0001	Significant
A—Printing speed	0.0002	1	0.0002	4.87	0.0476	Significant
B—Layer height	4.306 × 10^−6^	1	4.306 × 10^−6^	0.0910	0.7681	Non-significant
C—Exposure time	0.0057	1	0.0057	120.94	<0.0001	Significant
B^2^	0.0005	1	0.0005	11.46	0.0054	Significant
Residual	0.0006	12	0.0000	-	-	-
Lack of fit	0.0006	10	0.0001	8.76	0.1067	Non-significant
Pure Error	0.0000	2	6.333 × 10^−6^	-	-	-
Cor total	0.0072	16	-	-	-	-
*R*-squared	0.9213		Pred *R*-squared		0.8280	
Adj. *R*-squared	0.8950		Adeq precision		20.59	

**Table 8:** ANOVA table for the height of microneedles (mm) (Stat-Ease 360^®^).

Source	Sum of Squares	d*f*	Mean Square	*F*-Value	*p*-Value	Conclusion
Quadratic Model	0.6055	9	0.0673	73.38	<0.0001	Significant
B—Layer height	0.0117	1	0.0117	12.76	0.0091	Significant
C-Exposure time	0.1142	1	0.1142	124.58	<0.0001	Significant
B^2^	0.0118	1	0.0118	12.85	0.0089	Significant
C^2^	0.0080	1	0.0080	8.76	0.0211	Significant
Residual	0.0064	7	0.0009	-	-	-
Lack of fit	0.0062	5	0.0012	11.23	0.0838	Not significant
Pure Error	0.0002	2	0.001	-	-	-
Cor total	0.6119	16	-	-	-	-
*R*-squared	0.9895		Pred *R*-squared		0.8615	
Adj. *R*-squared	0.9760		Adeq precision		28.02	

**Table 9:** Dimensional accuracy results for each of the microneedle geometries prepared using the biocompatible 3D printed molds (Mean ± SD).

Geometry	Dimensional Evaluation		
Cone	**Lower Limit**	**Base Diameter:** Average: 270.6 ± 7.3 μm (90.2%)	**Height:** Average: 277.5 ± 4.7 μm (92.5%)
	**Upper Limit**	**Base Diameter:** Average: 474.0 ± 2.5 μm (94.8%)	**Height:** Average: 971.0 ± 12.6 μm (97.1%)
Inclined cone (tip at the edge)	**Medium Level**	**Base Diameter:** Average: 281.1 ± 5.1 μm (93.7%)	**Height:** Average: 606.5 ± 17.6 μm (93.3%)
Triangular pyramid	**Lower Limit**	**Base Height:** Average: 274.5 ± 6.6 μm (91.5%)	**Height:** Average: 263.7 ± 4.2 μm (87.9%)
	**Upper Limit**	**Base Height:** Average: 459.0 ± 11.0 μm (91.8%)	**Height:** Average: 985.0 ± 5.9 μm (98.5%)
Inclined triangular pyramid (tip at the edge)	**Medium Level**	**Base Height:** Average: 271.0 ± 4.2 μm (90.3%)	**Height:** Average: 549.3 ± 15.9 μm (84.5%)
Square pyramid	**Minimum Interspacing** **Distance**	**Interspacing Distance:** Average: 112.8 ± 3.5 μm (107.4%)	
	**Lower Limit**	**Base Width:** Average: 278.7 ± 5.5 μm (92.9%)	**Height:** Average: 288.9 ± 4.3 μm (96.3%)
	**Upper Limit**	**Base Width:** Average: 475.7 ± 9.9 μm (95.1%)	**Height:** Average: 987.4 ± 6.9 μm (98.7%)
Square pyramid with angled edges	**Lower Limit**	**Base Width:** Average: 273.4 ± 2.1 μm (91.1%)	**Height:** Average: 280.5 ± 5.3 μm (93.5%)
	**Upper Limit**	**Base Width:** Average: 463.5 ± 6.6 μm (92.7%)	**Height:** Average: 950.2 ± 9.5 μm (95.0%)
Hexagonal-based pyramid	**Lower Limit**	**Base Width:** Average: 275.4 ± 5.0 μm (91.8%)	**Height:** Average: 272.4 ± 9.0 μm (90.8%)
	**Upper Limit**	**Base Width:** Average: 470.5 ± 8.5 μm (94.1%)	**Height:** Average: 975.8 ± 5.9 μm (97.6%)
Beveled-tip	**Lower Limit**	**Base Diameter:** Average: 279.5 ± 3.5 μm (93.2%)	**Height:** Average: 274.8 ± 4.1 μm (91.6%)
	**Upper Limit**	**Base Diameter:** Average: 484.7 ± 3.8 μm (96.9%)	**Height:** Average: 990.8 ± 2.4 μm (99.1%)
Tapered-cone	**Lower Limit**	**Base Diameter:** Average: 271.8 ± 6.1 μm (90.6%)	**Height of Base:** Average: 139.8 ± 4.3 μm (93.0%) **Height of Cone:** Average: 142.1 ± 5.0 μm (94.7%)
	**Upper Limit**	**Base Diameter:** Average: 486.6 ± 3.7 μm (97.3%)	**Height of Base:** Average: 492.3 ± 4.2 μm (98.5%) **Height of Cone:** Average: 490.7 ± 3.9 μm (98.2%)
Obelisk	**Lower Limit**	**Base Width:** Average: 279.7 ± 3.2 μm (93.2%)	**Height of Base:** Average: 144.0 ± 1.3 μm (96.0%) **Height of Pyramid:** Average: 147.5 ± 1.6 μm (98.3%)
	**Upper Limit**	**Base Width:** Average: 485.1 ± 10.3 μm (97.0%)	**Height of Base:** Average: 482.6 ± 6.0 μm (96.5%) **Height of Pyramid:** Average: 489.5 ± 6.4 μm (97.9%)
Modified pyramid	**Lower Limit**	**Base Width:** Average: 236.8 ± 13.3 μm (78.9%)	**Height:** Average: 288.0 ± 6.1 μm (96.0%)
	**Upper Limit**	**Base Width:** Average: 461.5 ± 11.1 μm (92.3%)	**Height:** Average: 956.4 ± 26.8 μm (95.6%)
Arrow-like	**Lower Limit**	**Base Width:** Average: 283.4 ± 8.1 μm (94.5%)	**Height of Base:** Average: 147.8 ± 3.5 μm (98.5%) **Height of Pyramid:** Average: 149.3 ± 1.3 μm (99.5%)
	**Upper Limit**	**Base Width:** Average: 490.2 ± 4.0 μm (98.0%)	**Height of Base:** Average: 494.4 ± 2.5 μm (98.9%) **Height of Pyramid:** Average: 476.0 ± 14.28 μm (95.2%)

**Table 10:** Dimensional accuracy results of microneedle arrays fabricated for skin permeation studies (Mean ± SD).

Geometry	Dimensional Evaluation	
Square pyramid	**Base Width:** Average: 374.8 ± 6.9 μm (93.7%)	**Height:** Average: 644.8 ± 5.8 μm (99.2%)
Beveled-tip	**Base Diameter:** Average: 380.1 ± 3.1 μm (95.0%)	**Height:** Average: 632.3 ± 6.4 μm (97.3%)
Obelisk	**Base Width:** Average: 379.9 ± 10.5 μm (95.0%)	**Height of Base:** Average: 321.4 ± 3.0 μm (98.9%) **Height of Pyramid:** Average: 322.4 ± 1.9 μm (99.2%)

## Data Availability

Raw data supporting the conclusions of this study are available upon request from the authors.

## Abbreviations

The following abbreviations are used in this manuscript:

API(s)	Active Pharmaceutical Ingredient(s)
3D	Three-Dimensional
CAD	Computer-Aided Design
DoE	Design of Experiments
RopHCl	Ropinirole Hydrochloride
HPLC	High-Performance Liquid Chromatography
LCD	Liquid Crystal Display
FCCD	Face-Centered Central Composite Design
PVA	Polyvinyl alcohol
